# Taiwan Expert Consensus Recommendations for Switching to Aripiprazole Long-Acting Once-Monthly in Patients with Schizophrenia

**DOI:** 10.3390/jpm11111198

**Published:** 2021-11-13

**Authors:** Chih-Sung Liang, Tung-Ping Su, Ming-Hsien Hsieh, Chau-Shoun Lee, Joseph Kuo, Nan-Ying Chiu, Po-See Chen, Yung-Chieh Yen, Ya-Mei Bai

**Affiliations:** 1 Department of Psychiatry, Beitou Branch, Tri-Service General Hospital, Taipei 11243, Taiwan; lcsyfw@gmail.com; 2 Department of Psychiatry, National Defense Medical Center, Taipei 11490, Taiwan; 3 Department of Psychiatry, Cheng Hsin General Hospital, Taipei 11220, Taiwan; tomsu0402@gmail.com; 4 Department of Psychiatry, National Taiwan University Hospital, Taipei 10002, Taiwan; hsiehmh@ntu.edu.tw; 5 Department of Psychiatry, Mackay Memorial Hospital, Taipei 10449, Taiwan; csleepsyc@gmail.com; 6 Department of Psychiatry, Camillian Saint Mary’s Hospital Luodong, Yilan 26546, Taiwan; kuo2084@yahoo.com.tw; 7 Department of Psychiatry, Changhua Christian Hospital, Changhua 50006, Taiwan; 400786@cch.org.tw; 8 Department of Psychiatry, National Cheng Kung University Hospital, Tainan 704, Taiwan; chenps@mail.ncku.edu.tw; 9 Department of Psychiatry, E-Da Hospital, Kaohsiung 82445, Taiwan; jackycyen@yahoo.com; 10 Department of Psychiatry, Taipei Veterans General Hospital, Taipei 11217, Taiwan; 11 Department of Psychiatry, College of Medicine, National Yang Ming Chiao Tung University, Taipei 11221, Taiwan

**Keywords:** schizophrenia, long-acting injectable antipsychotic, aripiprazole, atypical antipsychotic, expert consensus, guidance, recommendation

## Abstract

In the last decade, long-acting injectable antipsychotics has been widely used in schizophrenia. Aripiprazole long-acting once-monthly (AOM) is the only long-acting dopamine partial agonist antipsychotic approved for schizophrenia; however, a literature search revealed no guidance on safely switching from oral and long-acting injectable antipsychotics to AOM. This study aimed to develop recommendations of AOM use based on existing data and expert consensus. A committee of 30 experts in psychopharmacology from major hospitals across Taiwan was invited. A modified Delphi method was conducted, consisting of two rounds of questionnaires, literature review, three rounds of face-to-face discussion meeting, and two rounds of anonymous voting. The consensus recommendations were developed based on existing data, clinical experiences, and consensus opinions, with 80% agreement among panel members required for final adoption. The panel developed nine consensus statements of switching to AOM for both acute and stable schizophrenia patients receiving oral or long-acting injectable atypical antipsychotics. Recommendations regarding dose adjustment of oral medication and pregnancy/breastfeeding were also included. The nine consensus recommendations provide a guidance on safely switching to AOM. Substantial gaps in knowledge, and more research is necessary.

## 1. Introduction

The most common cause of psychotic relapse in patients with schizophrenia is poor adherence to antipsychotics [1,2]. Long-acting injectable antipsychotics (LAIs) administered every 2 to 12 weeks can improve adherence compared to oral antipsychotics [3]. Aripiprazole long-acting once-monthly (AOM) is the first long-acting dopamine partial agonist antipsychotic approved for the treatment of schizophrenia [4]. Evidence has shown that AOM is effective in reducing relapse rates, and is associated with low rates of movement disorders and metabolic disturbances [4]. A recent network meta-analysis compared 12 LAIs and reported that AOM is ranked highly in effect size for both relapse prevention and tolerability [5].

Limited guidance exists for switching to AOM from other antipsychotics in patients with schizophrenia. A panel of 19 Italian and Spanish psychiatrists published a consensus for switching from a LAI to AOM for schizophrenia [6]; however, the consensus did not address switching from an oral antipsychotic to AOM. Another group of psychiatric experts in Hong Kong developed several consensus statements, aiming to facilitate the understanding and usage of aripiprazole. However, the consensus focused primarily on oral aripiprazole [7].

A committee of 30 psychopharmacological experts across Taiwan was convened. The aim of the committee was to combine the evidence with expert opinion to derive evidence- and consensus-based recommendations for switching to AOM in patients currently receiving other oral or LAI antipsychotics. The recommendations also covered pregnant and breastfeeding patients. We aimed to facilitate the understanding of clinical properties of AOM and provide practice-oriented recommendations for switching to AOM.

## 2. Materials and Methods

Our study utilized modified Delphi method [8] to generate expert consensus on recommendations for switching to AOM in patients with schizophrenia. The modified Delphi method consisted of two rounds of questionnaires, literature review, three rounds of face-to-face discussion meeting, and two rounds of anonymous voting (between 22 August 2019 and 18 August 2020).

Dr. Bai, as the President of the Taiwanese Society of Biological Psychiatry and Neuropsychopharmacology, invited 29 senior psychiatrists for an expert committee on switching to AOM via electronic mail or phone. The consensus committee included 30 senior psychiatrists and psychopharmacology experts (Table S1) from major hospitals across Taiwan, with at least three years of clinical experience in using AOM in both outpatient and inpatient settings.

After two rounds of questionnaires, the consensus committee focused on switching to AOM in patients with schizophrenia under nine various conditions (Table 1). This consensus started with the premise that acute patients have poor response to their current oral antipsychotics or LAIs, and thes stable patients are primarily switched to AOM to improve convenience or to reduce side effects, and not because of efficacy issues. Therefore, it was assumed that physicians have comprehensively assessed the patient and decided to switch to AOM due to potential benefits. It was also assumed that physicians could have access to both 300 mg and 400 mg dosage of AOM, although the committee acknowledges that this may not be the case for all hospitals or some countries. “Acute patients” were defined as patients with schizophrenia undergoing an acute psychotic episode, while “stable patients” refers to patients with schizophrenia whose symptoms are controlled or in remission.

A consensus structure was developed for literature review and recommendation development, and included information on the status of the patient (acute or stable) and current medication (Table 1). Committee members conducted a literature review across PubMed, Embase, the Cochrane Database of Systematic Reviews, and the Cochrane Central Register of Controlled Trials. Search terms included synonyms of (1) aripiprazole; (2) schizophrenia and related disorders, and (3) depot, (long-acting) injection(s), microsphere, decanoate, palmitate, enanthate, pamoate, and monohydrate. Studies published from database inception up to a last search on 31 July 2020 were evaluated. A narrative, rather than systematic, review was performed because of the thematic breadth of consensus statements and variability in the clinical setting and patient status. For example, we identified 18 studies in PubMed by using the above search terms and a filter for randomized controlled trials; however, none of the 18 studies could address the nine conditions. Therefore, committee members also assessed published guidelines and recent meta-analyses/systematic reviews to provide a comprehensive overview of the available evidence. Where the available evidence was insufficient, each committee member provided clinical experience and expert opinion pertaining to each proposed recommendation.

After the recommendations were developed, the consensus committee adopted a modified Delphi method with four rounds to evaluate each recommendation and the clinical practices described therein, including starting dosage, duration and dosage of concomitant oral medication, and other aspects of the switching process. Anonymous online voting was conducted to establish levels of agreement (LoA) for each recommendation, with the threshold for consensus set at 80%. For recommendations with <80% of LoA, the exact voting percentages for the practice options discussed are listed (Table 2). Eventually, nine recommendations were developed and approved by the consensus committee (Table 2). Equivalent doses to aripiprazole for oral atypical antipsychotics has been provided in Table 3 [9]. In Taiwan, the common oral atypical antipsychotics include aripiprazole, clozapine, olanzapine, quetiapine, and other serotonin-dopamine antagonist antipsychotics (SDAs) (namely, amisulpride, lurasidone, paliperidone, risperidone, and ziprasidone). The common LAI atypical antipsychotics are LAI aripiprazole, LAI olanzapine, LAI risperidone, and LAI paliperidone.

## 3. Results

The 30 experts had a mean of 23.87 (7.41) years of clinical experiences in treating schizophrenia (Table S1). The AOM constituted 38.87% (17.86) of LAI use among the 30 experts. Of these, 26/30 completed the round 1 questionnaire and 30/30 the round 2 questionnaire; 23/30 participated the round 1 face-to-face discussion meeting, 26/30 the round 2 meeting, and 30/30 the round 3 meeting; 26/30 completed the round 1 anonymous voting and 30/30 the round 2 voting. The consensus recommendations are listed below.

### 3.1. Recommendations

#### 3.1.1. Recommendation 1: Switching to AOM in Acute Patients from Oral Aripiprazole for Enhancing Treatment Effectiveness

- For acute patients on oral aripiprazole ≤15 mg, the recommended starting dose of AOM was 300 mg (LoA = 90%).
- Experts recommended maintaining the same dose of oral aripiprazole during the overlap period for acute patients receiving oral aripiprazole ≤15 mg (LoA = 80%).

This recommendation was based on the pharmacokinetic studies of steady state drug concentration in healthy volunteers receiving oral aripiprazole [10] and patients with schizophrenia receiving AOM [11], as well as the clinical experience of committee members. Experts recommended a starting dose of AOM 300 mg (LoA = 90%) and maintaining the same dose of oral aripiprazole during the overlap period (LoA = 80%) for acute patients on oral aripiprazole ≤15 mg. Importantly, only 17% of experts recommended ≤2 weeks of concomitant oral aripiprazole when switching to AOM from oral aripiprazole. For current oral dose of aripiprazole 16–20 mg/day, 67% of experts recommended maintaining the same dose of oral aripiprazole. For current oral dose of aripiprazole 20–30 mg/d, 73% of experts recommended reducing the dose of concomitant oral aripiprazole when switching to AOM.

#### 3.1.2. Recommendation 2: Switching to AOM in Acute Patients on Oral Atypical Antipsychotics (Excluding Aripiprazole and Clozapine) for Enhancing Treatment Effectiveness

- At least 3–7 days of oral aripiprazole was recommended to establish tolerability for aripiprazole-naïve patients (LoA = 100%).

A multicenter randomized controlled trial (RCT) has investigated the efficacy, tolerability, and safety of AOM vs. placebo in patients with schizophrenia (*n* = 340) [12]. In this RCT, patients without prior exposure to aripiprazole received open-label treatment with oral aripiprazole 10 mg/day for 3 days to establish tolerability prior to receiving AOM. Therefore, for aripiprazole-naïve patients, experts recommended at least 3–7 days of oral aripiprazole to establish tolerability before administering AOM (LoA = 100%). For patients who were being treated with other oral atypical antipsychotics, 67% of experts agreed that the starting dose of AOM should correspond to the current oral dose of atypical antipsychotics as converted to an equivalent dose of oral aripiprazole [13]; and the same was suggested for patients who could not tolerate their current oral atypical antipsychotics, in that their starting dose of oral aripiprazole should be equivalent to their oral dose of atypical antipsychotics (LoA = 63%).

For patients that could tolerate their current oral atypical antipsychotics, no consensus of concomitant antipsychotics was reached, with 43% of experts voting to maintain current oral atypical antipsychotics at the same dose, 27% voting to switch to oral aripiprazole at an equivalent dose, 17% voting to maintain current oral atypical antipsychotics at a lower dose, 10% voting to switch to oral aripiprazole at a lower dose, and 3% voting for other clinical practices. For the duration of overlap with oral atypical antipsychotics, 53% of experts suggested 5–12 weeks of overlap with current olanzapine, quetiapine, or aripiprazole when switching to AOM. No consensus was reached for patients receiving other SDAs (namely, amisulpride, lurasidone, paliperidone, risperidone, and ziprasidone).

One expert recommended that the duration of concomitant oral antipsychotics when switching to AOM should be based on pharmacokinetics [10]. Because the minimum duration to achieve the steady state concentration of AOM exceeds 12 weeks, the duration of overlap with oral atypical antipsychotics should preferably be at or greater than 12 weeks [9], and the overlap duration should not differ between any atypical antipsychotics. However, other experts pointed out that it was not necessary to reach steady state antipsychotic concentrations for symptom management, and the majority of experts agreed that in their clinical experience, olanzapine and quetiapine required a longer tapering time than other SDAs (namely, amisulpride, lurasidone, paliperidone, risperidone, and ziprasidone) for lowering the risk of rebound syndrome and rebound psychosis.

#### 3.1.3. Recommendation 3: Switching to AOM in Acute Patients on Oral Clozapine for Enhancing Treatment Effectiveness

- At least 3–7 days of oral aripiprazole is recommended to establish tolerability for aripiprazole-naïve patients (LoA = 100%).
- After successful switching to AOM (more than 4–5 doses), concomitant clozapine can be continued at a lower dose (LoA = 90%).

Evidence suggests that around 15–30% of patients with schizophrenia fail to respond to standard antipsychotic treatment and are considered to have treatment-resistant schizophrenia (TRS) [14,15,16]. Clozapine has been approved as an effective treatment for TRS [17]. However, a meta-analysis reported a 40% response rate to clozapine in patients with TRS, and suggested that 12–20% of people with TRS will be ultra-resistant [18]. A nationwide cohort study in Finland (*n* = 62,250) investigated 29 antipsychotics and reported that clozapine plus aripiprazole was associated with the lowest risk of psychiatric rehospitalization in the total cohort [19]; moreover, clozapine plus aripiprazole was superior to clozapine, which was considered to be the best antipsychotic monotherapy in the entire cohort [19]. A meta-analysis investigating augmentation strategies for clozapine refractory schizophrenia also reported that aripiprazole is the most effective antipsychotic augmentation agent for this population [20]. Taken together, the available evidence suggests that combining aripiprazole with clozapine may be useful in the treatment of schizophrenia.

Committee members stated that it could be challenging to achieve symptom control or taper concomitant oral clozapine in acute patients augmented with AOM, and therefore it was recommended that AOM should be started at 400 mg, while maintaining oral clozapine at the original dose during the first 4–5 doses of AOM treatment (LoA = 90%).

#### 3.1.4. Recommendation 4: Switching to AOM in Stable Patients on Oral Aripiprazole for Enhancing Treatment Effectiveness

- For stable patients on oral aripiprazole ≤10 mg, the recommended starting dose of AOM was 300 mg (LoA = 97%).
- For stable patients on oral aripiprazole of 11–15 mg (LoA = 57%) and of 16–30 mg (LoA = 80%), the majority of experts recommended decreasing the dose of aripiprazole when switching to AOM.

When switching to AOM from oral aripiprazole, the majority of experts recommended that the dose of concomitant oral aripiprazole in stable patients should be lower than that for acute patients (Recommendation 1). However, no consensus was achieved on the duration of concomitant oral aripiprazole when switching to AOM, with 43% of experts voting for 3–4 weeks of overlap with oral aripiprazole, 37% voting for ≤2 weeks, 17% voting for 5–12 weeks, and 3% voting for ≥13 weeks. Some experts favoured a longer overlap period (i.e., >12 weeks) of oral aripiprazole to reduce the risk of relapse. This is because the median time for relapse occurrence after stopping LAIs was about two months, and may be associated with inability to maintain steady state drug concentrations.

#### 3.1.5. Recommendation 5: Switching to AOM in Stable Patients on Oral Atypical Antipsychotics (Excluding Aripiprazole and Clozapine) for Enhancing Treatment Effectiveness

- At least 3–7 days of oral aripiprazole is recommended to establish tolerability for aripiprazole-naïve patients (LoA = 100%).
- The starting dose of AOM should correspond to the current oral dose of atypical antipsychotics as converted to an equivalent dose of oral aripiprazole (LoA = 80%).

In stable patients on oral olanzapine, quetiapine, or other SDAs (namely, amisulpride, lurasidone, paliperidone, risperidone, and ziprasidone), no consensus was reached for the duration of overlap with concomitant oral atypical antipsychotics during switching to AOM. However, the majority of experts (LoA = 53%) recommended maintaining 3–4 weeks of concomitant oral atypical antipsychotics for patients receiving other SDAs (namely, amisulpride, lurasidone, paliperidone, risperidone, and ziprasidone) and maintain, and 57% of experts recommended 5–12 weeks of concomitant oral atypical antipsychotics for patients receiving quetiapine or olanzapine. For stable patients with good tolerability of current atypical antipsychotics, 47% of experts voted to maintain the current oral medication at a lower dose when switching to AOM, 30% voted to maintain the current oral medication at the same dose, 13% voted to switch to oral aripiprazole at a lower equivalent dose, and 10% voted to switch to oral aripiprazole at an equivalent dose (Table 3). If stable patients did not tolerate their current oral atypical antipsychotics, 47% of experts voted to switch to oral aripirazole at a lower equivalent dose, 40% voted to switch to oral aripiprazole at an equivalent dose, and 13% voted to maintain the original oral medication at a lower dose.

#### 3.1.6. Recommendation 6: Switching to AOM in Stable Patients on Oral Clozapine for Enhancing Treatment Effectiveness

- At least 3–7 days of oral aripiprazole is recommended to establish tolerability for aripiprazole-naïve patients (LoA = 97%).
- After successful switching to AOM (more than 4–5 doses), concomitant clozapine can be continued at a lower dose (LoA = 97%).

For stable patients on oral clozapine switching to AOM, the consensus and recommendations were similar to those for acute patients (Recommendation 3). Either acute patients or stable patients, the majority of experts recommended starting AOM at a dose of 400 mg and maintaining oral clozapine at the original dose as concomitant medication during the first 4–5 doses of AOM treatment.

#### 3.1.7. Recommendation 7: Switching to AOM in Acute Patients on other LAIs for Enhancing Treatment Effectiveness

- At least 3–7 days of oral aripiprazole is recommended to establish tolerability for aripiprazole-naïve patients (LoA = 100%).
- When switching from 1-month LAI paliperidone palmitate to AOM, concomitant oral aripiprazole for at least 2 weeks is recommended (LoA = 80%).

When switching to AOM, the majority of experts recommended the original LAI could be stopped without dose tapering (LoA = 67%), and AOM should be initiated prior to the next dose of the original LAI (LoA = 67%). Besides, the recommended starting dose of AOM was 400 mg (LoA = 63%). The majority of experts (range of LoA: 67–80%) recommended concomitant oral aripiprazole medications for at least 2 weeks but not original LAI corresponding oral formulation. For example, when switching from 3-month LAI paliperidone palmitate to AOM, 3% of experts voted oral paliperidone for at least 2 weeks.

#### 3.1.8. Recommendation 8: Switching to AOM in Stable Patients on Other LAIs for Enhancing Treatment Effectiveness

- At least 3–7 days of oral aripiprazole is recommended to establish tolerability for aripiprazole-naïve patients (LoA = 100%).

Recommendations for switching from other LAIs to AOM were primarily based on a 2016 expert consensus by a panel of Italian and Spanish psychiatrists [6], the Taiwan NHI reimbursement regulations, and the clinical experience of committee members. The majority of experts recommended directly switching to AOM to replace the next dose of the original LAI in stable patients; however, in acute patients, AOM should be administered prior to the next dose of the original LAI. Concomitant oral aripiprazole for at least two weeks was recommended for both acute and stable patients. However, the recommended starting dose of AOM should be based on the dose of the original LAI equivalent oral aripiprazole dose (Table 3).

#### 3.1.9. Recommendation 9: For Pregnant or Lactating Patients on AOM

- The majority of experts recommended that if the benefits of continuing treatment outweigh the risks, AOM could be continued for pregnant patients after education of the potential risks (LoA = 80%).
- The majority of experts recommended that if the benefits of continuing treatment outweigh the risks, AOM could be continued for lactating patients after education of the potential risks (LoA = 80%).

Women with schizophrenia had higher risk of adverse obstetric and neonatal outcomes than the general population, such as low birthweights or preterm delivery [20]. The potential mechanisms may include schizophrenia itself, an unhealthy lifestyle, comorbid physical conditions, and social adversities [21]. Whether psychotropics increase the risk of the adverse obstetric and neonatal outcomes remains inconclusive because of limited data on untreated pregnancy women with schizophrenia. Evidence from studies using untreated women with schizophrenia as a comparison group reported few associations between antipsychotics and adverse obstetric and neonatal outcomes [22,23,24]. On the other hand, little published evidence exists with regard to the safety of oral aripiprazole during breastfeeding. Therefore, the experts did not achieve a consensus for this recommendation, and the risk and benefit of AOM needs to be evaluated individually. However, the majority of experts agreed that the benefits of breastfeeding likely outweigh the harmful effects of oral aripiprazole. Therefore, if the benefits of continuing treatment outweigh the risks, AOM is recommended to be continued for pregnant or lactating patients after education of the potential risks. However, some experts stated that they would consider switching to oral aripiprazole due to a shorter half-life. The consensus committee agreed that the risks of treatment should be fully communicated to patients in an understandable way, and AOM treatment requires more intensive monitoring of the mother and fetus/infant during pregnancy and lactation.

## 4. Discussion

Many factors (e.g., patient conditions, clinical settings, and other extraneous factors) might affect switching strategies, and the consensus committee established a set of premises to converge the discussion and decision-making process. The committee acknowledges that clinical practice may not be as clear-cut, and these recommendations may be subject to change as new evidence becomes available; therefore, the attending physician should have ultimate discretion over the switching process. The reasons for switching, potential benefits and risks, switching strategy, and monitoring must be clearly communicated in an easily understandable way to patients, caregivers, and family members at every step of the switching process, to avoid relapses and to optimize patient outcomes.

The current study has limitations. First, the literature review did not find any randomized control trials (RCTs) addressing switching from oral or long-acting injectable antipsychotics to AOM. Therefore, all the consensus recommendations were not based on RCT evidence. Second, all exerts invited are from Taiwan, and there might be limitations on the representatives of the clinical experiences and hence the generalizability of the recommendations. Third, we did not discuss oral and LAI typical antipsychotics.

However, the recommendations can still offer insight from experts’ clinical experience, and may serve as a reference for daily practice and a guide to design future observational studies or RCTs to fill the gaps in AOM switching research. As more clinical evidence and experience becomes available, the consensus committee expects to update these recommendations accordingly, and it is also hoped that expert opinions will gradually consolidate around a stronger consensus of clinical practices. In addition, input from patients and caregivers may also be brought in during future revisions to include additional perspectives and concerns.

## 5. Conclusions

Safely switching from oral to long-acting antipsychotics is particularly relevant in clinical practice. AOM and other LAIs can bring many benefits in terms of adherence, symptom control, safety, and quality of life for patients with schizophrenia. The development of best practices that take clinical circumstances into account can help patients to make the most of such benefits, and it is hoped that better care and treatment outcomes will eventually reduce the stigma associated with mental disease in society today.

## Figures and Tables

**Table 1:** Structure of the Consensus Recommendations.

Patient Status	Current Medication	Consensus Recommendation
Acute	Oral aripiprazole	Recommendation 1
	Oral atypical antipsychotics (excluding aripiprazole and clozapine)	Recommendation 2
	Oral clozapine	Recommendation 3
Stable	Oral aripiprazole	Recommendation 4
	Oral atypical antipsychotics (excluding aripiprazole and clozapine)	Recommendation 5
	Oral clozapine	Recommendation 6
Acute	Long-acting injectable antipsychoitcs	Recommendation 7
Stable	Long-acting injectable antipsychotics	Recommendation 8
Pregnant or Lactating	Aripiprazole long-acting once-monthly	Recommendation 9

**Table 2:** Consensus Recommendations.

**Recommendation 1: Switching to AOM in Acute Patients from Oral Aripiprazole for Enhancing Treatment Effectiveness.**	**Practice Option**	**LoA**
Treatment initiation with AOM 300 mg is recommended for patients whose current dose of oral aripiprazole is lower than 15 mg.		90%
The majority of experts recommend maintaining 3–4 weeks of concomitant oral aripiprazole treatment when switching to AOM.	3–4 weeks	53%
	5–12 weeks	23%
	≤2 weeks	17%
	≥13 weeks	7%
For patients currently receiving an oral aripiprazole dose of ≤15 mg, it is recommended to maintain the same dose level for concomitant oral aripiprazole treatment when switching to AOM.		80%
For patients currently receiving an oral aripiprazole dose of 16–20 mg, the majority of experts recommend maintaining the same dose level for concomitant oral aripiprazole treatment when switching to AOM.	Maintain	67%
	Decrease	33%
For patients currently receiving an oral aripiprazole dose of 21–30 mg, the majority of experts recommend decreasing the same dose level for concomitant oral aripiprazole treatment when switching to AOM.	Decrease	73%
	Maintain	27%
**Recommendation 2: Switching to AOM in acute patients on oral atypical antipsychotics (excluding aripiprazole and clozapine) for enhancing treatment effectiveness.**	**Practice Option**	**LoA**
At least 3–7 days of prior treatment with oral aripiprazole is recommended for patients who have never been treated with aripiprazole before.		100%
The majority of experts recommend that the starting dose of AOM should be based on the oral aripiprazole dose equivalent to the current dose of oral atypical antipsychotics (please see Table 3).	Based on equivalent oral aripiprazole dose	67%
	AOM 400 mg preferred	33%
The majority of experts recommend that for patients who cannot tolerate their current oral atypical antipsychotics (excluding aripiprazole and clozapine), they should be switched to oral aripiprazole at a dose equivalent to the current dose of oral atypical antipsychotics (please see Table 3) for concomitant oral therapy when switching to AOM.	Switch to equivalent oral aripiprazole dose	63%
	Maintain original oral atypical antipsychotics but at reduced dose	23%
	Switch to lower oral aripiprazole dose	10%
The majority of experts recommend maintaining 5–12 weeks of concomitant oral atypical antipsychotics (maintaining original olanzapine or quetiapine, or switching to aripiprazole) for patients receiving quetiapine or olanzapine.	5–12 weeks	53%
	≥13 weeks	23%
	3–4 weeks	20%
	Other	3%
**Recommendation 3: Switching to AOM in acute patients on oral clozapine for enhancing treatment effectiveness.**	**Practice Option**	**LoA**
At least 3–7 days of prior treatment with oral aripiprazole is recommended for patients who have never been treated with aripiprazole before.		100%
The majority of experts recommended starting AOM at a dose of 400 mg.	400 mg	77%
	Based on clozapine equivalent oral aripiprazole dose	20%
	300 mg	3%
The majority of experts recommended maintaining oral clozapine at the original dose as concomitant medication during the first 4–5 doses of AOM treatment.	Oral clozapine at original dose	77%
	Oral clozapine at reduced dose	23%
For patients that have achieved stable treatment (more than 4–5 doses) after switching to AOM, concomitant oral medication with clozapine can be continued at a reduced dose.		90%
**Recommendation 4: Switching to AOM in stable patients on oral aripiprazole for enhancing treatment effectiveness.**	**Practice Option**	**LoA**
Treatment initiation with AOM 300 mg is recommended for patients whose current dose of oral aripiprazole is lower than 10 mg.		97%
For patients currently receiving an oral aripiprazole dose of ≤10 mg, the majority of experts recommend maintaining the same dose level for concomitant oral aripiprazole treatment when switching to AOM.	Maintain	57%
	Decrease	43%
For patients currently receiving an oral aripiprazole dose of 11–15 mg, the majority of experts recommend decreasing the dose level for concomitant oral aripiprazole treatment when switching to AOM.	Decrease	57%
	Maintain	43%
For patients currently receiving an oral aripiprazole dose of 16–30 mg, it is recommended to decrease the dose level for concomitant oral aripiprazole treatment when switching to AOM.		80%
**Recommendation 5: Switching to AOM in stable patients on oral atypical antipsychotics (excluding aripiprazole and clozapine) for enhancing treatment effectiveness.**	**Practice Option**	**LoA**
At least 3–7 days of prior treatment with oral aripiprazole is recommended for patients who have never been treated with aripiprazole before.		100%
The starting dose of AOM should be based on the oral aripiprazole dose equivalent to the current dose of oral atypical antipsychotics (please see Table 3).		80%
The majority of experts recommend maintaining 3–4 weeks of concomitant oral atypical antipsychotics (maintaining original other SDAs or switching to aripiprazole) for patients receiving other SDAs (namely, amisulpride, lurasidone, paliperidone, risperidone, and ziprasidone).	3–4 weeks	53%
	≤2 weeks	30%
	5–12 weeks	13%
	≥13 weeks	3%
The majority of experts recommend maintaining 5–12 weeks of concomitant oral atypical antipsychotics (maintaining original olanzapine or quetiapine, or switching to aripiprazole) for patients receiving quetiapine or olanzapine.	5–12 weeks	57%
	3–4 weeks	27%
	≥13 weeks	17%
**Recommendation 6: Switching to AOM in stable patients on oral clozapine for enhancing treatment effectiveness.**	**Practice Option**	**LoA**
At least 3–7 days of prior treatment with oral aripiprazole is recommended for patients who have never been treated with aripiprazole before.		97%
The majority of experts recommended starting AOM at a dose of 400 mg.	400 mg	63%
	Based on clozapine equivalent oral aripiprazole dose	27%
	300 mg	10%
The majority of experts recommended maintaining oral clozapine at the original dose as concomitant medication during the first 4–5 doses of AOM treatment.	Oral clozapine at original dose	77%
	Oral clozapine at reduced dose	23%
For patients that have achieved stable treatment (more than 4–5 doses) after switching to AOM, concomitant oral medication with clozapine can be continued at a reduced dose.		97%
**Recommendation 7: Switching to AOM in acute patients on other LAIs for enhancing treatment effectiveness.**	**Practice Option**	**LoA**
At least 3–7 days of prior treatment with oral aripiprazole is recommended for patients who have never been treated with aripiprazole before.		100%
The majority of experts recommended that the original LAI should be stopped upon initiating AOM treatment.	Stopped upon initiation of AOM	67%
	Tapered following stable AOM use	33%
The majority of experts recommended that AOM should be initiated prior to the next dose of the original LAI	Prior to the next dose	67%
	Replace the next dose	33%
The majority of experts recommended starting AOM at a dose of 400 mg.	400 mg	63%
	Based on the dose of the original LAI	37%
When switching from LAI risperidone (Risperdal Consta^®^) to AOM, the majority of experts recommended concomitant oral aripiprazole medication for at least 2 weeks.	Oral aripiprazole for at least 2 weeks	67%
	No concomitant oral medication needed	17%
	Oral risperidone for at least 2 weeks	13%
	Other	3%
When switching from 1-month LAI paliperidone palmitate (Invega Sustenna^®^) to AOM, concomitant oral aripiprazole medication for at least 2 weeks is recommended.		80%
When switching from 3-month LAI paliperidone palmitate (Invega Trinza^®^) to AOM, the majority of experts recommended concomitant oral aripiprazole medication for at least 2 weeks.	Oral aripiprazole for at least 2 weeks	70%
	No concomitant oral medication needed	27%
	Oral paliperidone for at least 2 weeks	3%
**Recommendation 8: Switching to AOM in stable patients on other LAIs for enhancing treatment effectiveness.**	**Practice Option**	**LoA**
At least 3–7 days of prior treatment with oral aripiprazole is recommended for patients who have never been treated with aripiprazole before.		100%
The majority of experts recommended that the original LAI should be stopped upon initiating AOM treatment.	Stopped upon initiation of AOM	77%
	Tapered following stable AOM use	23%
The majority of experts recommended that AOM should be initiated to replace the next dose of the original LAI	Replace the next dose	60%
	Prior to the next dose	40%
The majority of experts recommended that the starting dose of AOM should be based on the dose of the original LAI (please see Table 3).	Based on the dose of the original LAI	77%
	400 mg	23%
When switching from LAI risperidone (Risperdal Consta^®^) to AOM, the majority of experts recommended concomitant oral aripiprazole medication for at least 2 weeks.	Oral aripiprazole for at least 2 weeks	63%
	No concomitant oral medication needed	30%
	Oral risperidone for at least 2 weeks	7%
When switching from 1-month LAI paliperidone palmitate (Invega Sustenna^®^) to AOM, the majority of experts recommended concomitant oral aripiprazole medication for at least 2 weeks.	Oral aripiprazole for at least 2 weeks	70%
	No concomitant oral medication needed	27%
	Oral paliperidone for at least 2 weeks	3%
When switching from 3-month LAI paliperidone palmitate (Invega Trinza^®^) to AOM, the majority of experts recommended concomitant oral aripiprazole medication for at least 2 weeks.	Oral aripiprazole for at least 2 weeks	53%
	No concomitant oral medication needed	47%
**Recommendation 9: For pregnant or lactating patients on AOM.**		
As the benefits of continuing treatment outweigh the risks, continued use of AOM is recommended for pregnant patients, and in addition to continuous monitoring, patients should be informed about relevant risks.		80%
As the benefits of continuing treatment outweigh the risks, continued use of AOM is recommended for lactating patients, and in addition to continuous monitoring, patients should be informed about relevant risks.		80%

**Abbreviation:** AOM = aripiprazole long-acting once-monthly; LAI = long-acting injectable.

**Table 3:** Dose Equivalencies (Risperidone 1 mg) for Common Oral Antipsychotics in Taiwan.

Medication	Daily Equivalent Dose
Oral antipsychotics	
Amisulpride	85.77 mg
Aripiprazole	1.84 mg
Clozapine	Not available
Lurasidone	23.49 mg
Olanzapine	2.42 mg
Paliperidone	2.13 mg
Quetiapine	77.01 mg
Risperidone	1.00 mg
Ziprasidone	29.77 mg/d
Long-acting injectable antipsychotics (LAI)	
LAI Olanzapine	3.16 mg
LAI Paliperidone	1.53 mg
LAI Risperidone	0.42 mg

Dose equivalents are based on the minimum effective dose method (risperidone 1 mg equivalent) [9].

## Data Availability

Data are available on reasonable request from the corresponding author.

## Supplementary Materials

Supplementary Materials: The following are available online at https://www.mdpi.com/article/10.3390/jpm11111198/s1, Table S1: Characteristics and clinical experiences of 30 experts in the field of psychopharmacology.
