# Supplementary material for: Taiwan Expert Consensus Recommendations for Switching to Aripiprazole Long-Acting Once-Monthly in Patients with Schizophrenia
Source: J Pers Med. 2021 Nov 13;11(11):1198. doi: 10.3390/jpm11111198 (PMC8621108; doi:10.3390/jpm11111198)
Supplement: Supplementary file 1 [file jpm-11-01198-s001.zip › jpm-1426837-supplementary.pdf]

Table S1. Characteristics and clinical experiences of 30 experts in the field of psychopharmacology.

	Name	Institution	Degree	Clinical Experience in Treating Schizophrenia (y)	Mean Number of Treating Schizophrenia (m)	Proportion of Patients with Schizophrenia Treated with Antipsychotics (%)	Proportion of Prescription of Long-Acting Antipsychotics (%)	Proportion of AOM when Using Long-Acting Antipsychotics (%)
1	Bo-Chung Chu	Chung Shan Medical University Hospital	Lecturer	20	250	95%	45%	50%
2	Chao Huang	Wei Gong Memorial Hospital	Medical doctor	30	100	95%	15%	40%
3	Chau-Shoun Lee	Mackay Memorial Hospital	Associate professor	33	65	99%	30%	40%
4	Chi-Fa Hung	Kaohsiung Chang Gung Memorial Hospital	Assistant professor	21	150	100%	10%	40%
5	Chih-Cheng Chang	Chi Mei Medical Center	Assistant professor	19	80	100%	30%	30%
6	Chih-Sung Liang	Tri-Service General Hospital Beitou Branch	Assistant professor	14	500	100%	20%	50%
7	Chin-Bin Yeh	Tri-Service General Hospital	Professor	28	300	100%	25%	50%
8	Ching-Yen Chen	Keelung Chang Gung Memorial Hospital	Associate professor	25	80	100%	40%	60%
9	Fong-Gang Wang	Changhua Christian Hospital	Medical doctor	25	150	100%	15%	40%
10	Huang-Chi Lin	Kaohsiung Medical University Chung-Ho Memorial Hospital	Assistant professor	19	160	100%	35%	50%
11	Hung-Chieh Wu Chang	National Taiwan University Hospital Yunlin Branch	Medical doctor	20	120	99%	20%	55%
12	Joseph Kuo	Camillian Saint Mary's Hospital Luodong	Medical doctor	26	250	99%	20%	80%
13	Kao-Ching Chen	National Cheng Kung University Hospital	Associate professor	20	120	100%	20%	50%

14	Li-Chung Huang	Taichung Veterans General Hospital Chiayi Branch	lecturer	13	450	98%	30%	75%
15	Linen Lin	En Chu Kong Hospital	Associate professor	23	120	100%	40%	30%
16	Ming-Hsien Hsieh	National Taiwan University Hospital	Associate professor	23	200	100%	50%	35%
17	Nan-Ying Chiu	Changhua Christian Hospital	Assistant professor	35	250	99%	25%	30%
18	Po-See Chen	National Cheng Kung University Hospital	Professor	22	50	99%	50%	30%
19	Sang-Wen Chang	Shin Kong Wu Ho Su Memorial Hospital	Assistant professor	30	60	100%	15%	34%
20	Shang-Chien Huang	Tungs' Taichung MetroHarbor Hospital	lecturer	20	200	100%	25%	40%
21	Tung-Ping Su	Cheng Hsin General Hospital	Professor	46	12	100%	35%	17%
22	Ya-mei Bai	Taipei Veterans General Hospital	Professor	30	150	100%	20%	15%
23	Yau Hung	Mennonite Christian Hospital	Medical doctor	34	200	99%	10%	50%
24	Yen-Jung Chen	Lotung Poh-Ai Hospital	Medical doctor	16	200	99%	40%	20%
25	Yi-Ju Pan	Far Eastern Memorial Hospital	Associate professor	20	150	95%	25%	15%
26	Ying-Jay Liou	Taipei Veterans General Hospital	Assistant professor	20	150	90%	15%	50%
27	Yu- Shian Cheng	Tsyr-Huey Mental Hospital	lecturer	10	300	100%	80%	10%
28	Yu-Chi Yeh	Cathay General Hospital	Medical doctor	26	90	100%	30%	50%
29	Yu-Chih Shen	Hualien Tzu Chi Hospital	Associate professor	20	35	99%	15%	40%
30	Yung-Chieh Yen	E-Da Hospital	Professor	28	50	100%	35%	40%

Supplementary Materials: The following are available online at www.mdpi.com/xxx/s1, Table S1: Characteristics and clinical experiences of 30 experts in the field of psychopharmacology.
